# B‐class gene GLOBOSA – a facilitator for enriched species diversity of *Salvia* in the New World?

**DOI:** 10.1111/plb.70002

**Published:** 2025-02-18

**Authors:** S. Wetters, P. Nick

**Affiliations:** ^1^ Department of Molecular Cell Biology Joseph Gottlieb Kölreuter Institute of Plant Science (JKIP), Karlsruhe Institute of Technology Karlsruhe Germany

**Keywords:** Biodiversity, evolution, flower, *GLOBOSA*, *Salvia*, speciation

## Abstract

- The genus *Salvia*, comprising around 1000 species, half of which are found in the New World, belongs to the taxonomically most challenging genera within the Lamiaceae. A part of this diversity can be ascribed to the shape and expansion of the corolla and stamen structures, because changes in geometry of the sexual organs and attractance of pollinators might establish propagation barriers. However, the structural, functional, and evolutionary context of the underlying genes has not yet been elaborated.
- In this study, we analyse a large set of flowers from *Salvia* species of different geographic origin and use this morphometric framework to address gene expression and phylogenetic analysis of the MADS‐box B‐class gene, *GLOBOSA*.
- We examined expression of *GLOBOSA* in petals and anthers throughout anthesis for both *Salvia pratensis* L., as species from Europe, and the American *Salvia elegans* Vahl. Structural analysis of the B‐class genes reveals typical MADS‐MIKC‐type composition. When we infer phylogenies for *GLOBOSA* and its binding partner *DEFICIENS*, we see a genus‐wide duplication of *DEFICIENS* in *Salvia* and a specific duplication of *GLOBOSA* in *Salvia* species from the New World.
- Based on the first description of flowering genes in the genus *Salvia*, we arrive at a working model, where a duplication of *GLOBOSA* enabled the intense radiation of New World *Salvia* by neo‐functionalization of a flower identity gene for morphogenetic control of corolla and anther geometry. We propose that the genus *Salvia* can be used as paradigm to address the role of EvoDevo for plant speciation.

## Graphical abstract

Analysis of B‐class genes of flowering in *Salvia* revealed a duplication of *DEFICIENS* in the whole genus and a geographically restricted duplication of *GLOBOSA* in the New World, correlating with enhanced species richness in the Americas.

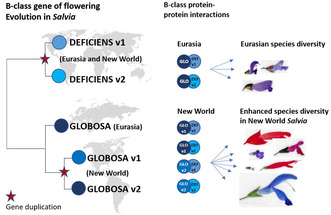

## INTRODUCTION

With around 1000 species, *Salvia* is among the most differentiated genera within the Lamiaceae. Several species have aromatic and medicinal properties, such as common sage (*Salvia officinalis* L.), others have attractive flowers as ornamental plants (e.g., *Salvia splendens*, *Salvia patens* Cav., *Salvia farinacea* Benth.). Two of the original four stamina that are typical for the tribe Mentheae have been functionally lost (Walker & Sytsma 2007), while the remaining stamina have been modified into lever‐like structures, representing a “key innovation” for this genus (Claßen‐Bockhoff *et al*. 2004). The genetic basis for this staminal lever has remained elusive. Moreover, it is unclear to what extent the lever mechanism can be considered as synapomorphy in the genus *Salvia*. In fact, this genus has been proposed as paraphyletic, with the genera *Rosmarinus, Perovskia, Zhumeria, Meriandra*, and *Dorystaechas* (in total 15 species) disrupting the monophyly of *Salvia sensu lato* (Walker *et al*. 2004; WWill & Claßen‐Bockhoff 2017). Other authors integrate these taxa into genus *Salvia*, including renaming e.g., *Rosmarinus officinale* as *Salvia rosmarinus* (Drew *et al*. 2017).

Many recent studies have increased understanding of the biogeographic history and evolution of genus *Salvia*, although questions remain. Large‐scale phylogenetic analysis including 519 species revealed four shifts in species diversification rates in the genus, but these rates are not strongly correlated with shifts in biome or pollinator system (Kriebel *et al*. 2019). The radiation of New World *Salvia* subgenus *Calosphace* may be ascribed to an initial shift to hummingbird pollination, followed by several reversals to insect pollination (Kriebel *et al*. 2019). However, this was challenged by arguing that anther connective and style shapes are not associated with shifts to hummingbird pollination, but rather are the cause of contingent evolution (Sazatornil *et al*. 2023). For flowering plants in general and for *Salvia* in particular, the geometry of flower organs controls reproductive isolation, as illustrated by the sympatric Old World *Salvia* species in Austria that deposit pollen at different sites on the pollinator, from where it is picked up by the style of the recipient (Claßen‐Bockhoff *et al*. 2004). The functionality of the staminal lever mechanism depends on shape of corolla, anther connective, and style (Kriebel *et al*. 2020), but shape of the stigma might also drive evolutionary processes towards switches in pollination system (Kriebel *et al*. 2022). In any case, the geometry of the flower depends on the genes controlling flower morphogenesis.

Homeobox transcription factors responsible for the identity of flower organs have received most attention, culminating in the well‐known ABC model of flowering, which provided an elegant explanation for phenotypes of homeotic mutants, where organs in one floral whorl are replaced by organs normally found in a different whorl (Bowman *et al*. 1991; Coen & Meyerowitz 1991). Later, the discovery of D‐class genes, responsible for ovule development, and E‐class genes, required for the transition to a floral meristem, extended the model to a mechanism, where hetero‐tetramers of transcription factors encoded by the ABCDE genes define the identity of the different flower organs (Theissen & Saedler 2001). The encoded proteins are composed of a DNA‐binding MADS domain (M), an intervening domain (I), the keratin‐like domain (K), and a C‐terminal domain, such that this subclade of MADS domain proteins is also known as MIKC (for review see Theißen *et al*. 2016). Among the first identified MIKC proteins were the so‐called B‐class factors, which are expressed in the central two whorls of the flower. Their loss‐of‐function results in a spectacular phenotype with a homeotic transformation of petals and anthers. The molecular explanation for this phenotype in the model organisms *Anthirrhinum majus* and *Arabidopsis thaliana* became one of the most convincing cornerstones supporting general acceptance of the ABC model.

Interestingly, in both of the above model plants the B‐class function is conveyed by two different genes: for *An. majus DEFICIENS* and *GLOBOSA* (Sommer *et al*. 1990; Tröbner *et al*. 1992), for *Ar. thaliana APETALA3* and *PISTILLATA* (Jack *et al*. 1992; Goto & Meyerowitz 1994). Again, in both model plants the respective gene products act as heterodimers (McGonigle *et al*. 1996; Melzer *et al*. 2014). The evolutionary history of this hetero‐dimerization seems to be ancient and has been proposed to date back to the Most Recent Common Ancestor of the Angiosperms. However, there is evidence for homodimers of both *GLO* and *DEF* in early Angiosperms (Melzer *et al*. 2014). These concurrent dimers shifted out of use, first the *DEF* homodimers, later the *GLO* homodimers. Interestingly, expression of both B‐class genes is not limited to petals and stamina in basal Angiosperms, such as the Magnoliids (Kim *et al*. 2005). The origin of the core eudicots was marked by a duplication of *DEF* (Zahn *et al*. 2005), such that the core eudicots are endowed with at least three B‐class genes. In many core eudicots a further gene duplication even added a third *DEF* gene, providing ample space for neo‐functionalization (Galimba *et al*. 2018). This might be the reason why phylogenies based on B‐class genes often deviate from phylogenies inferred from morphological traits, even in well‐known monophyletic taxa (Zahn *et al*. 2005). B‐class genes, therefore, seem to be ideal candidates for application to a genus with highly diverse corolla and stamen structures.

We wondered whether the obviously elusive taxonomy of *Salvia*, together with their vast floral diversity, might be linked to potential duplications and neo‐functionalizations of B‐class genes of flowering. Therefore, we applied a holistic approach to this genus: (i) morphology of flower organs was phenotyped quantitatively and dissected using Principal Components Analysis (PCA); (ii) expression of floral identity genes was followed through flower development; and (iii) a phylogeny of genus *Salvia* was constructed on the basis of B‐class genes. This EvoDevo strategy revealed that a duplication of *GLO* represents a synapomorphy of New World *Salvia*.

## MATERIAL AND METHODS

### Plant material

This study employed 42 accessions of *Salvia*: 26 accessions from Europe, 12 from America, and four from Asia. As outgroups, two accessions of *Dracocephalum*, and one accession each from the genera *Ocimum*, *Lavandula*, and *Sesamum* were included. Table 1 provides details on these accessions, voucher codes under which they are accessible at the collection of KIT‐JKIP, and GenBank accession numbers of the sequences recovered from them. To obtain a reliable framework for all subsequent (morphological and molecular) investigations, we verified the taxonomic identity of our reference plants using morphology‐based determination keys (Hegi 1927; Brach & Song 2006; Schmeil & Fitschen 2011).

**Table 1:** *Salvia* accessions in this study.

voucher ID – KIT	species taxon	origin	subgenus Kriebel *et al*., 2019	subgenus Claßen‐Bockhoff *et al*.
83	*Salvia officinalis* L.	Europe	*Salvia*	I‐D Salvia s.s
478	*Salvia tomentosa* Mill.	Europe	*Salvia*	I‐D Salvia s.s
1482	*Salvia miltiorrhiza* Bunge	Asia	*Glutinaria*	IV‐B Glutinaria
4680	*Rosmarinus officinalis* L. *Salvia rosmarinus*	Europe	*Rosmarinus*	Rosmarinus
4684	*Salvia lavandulifolia* Vahl.	Europe	*Salvia*	I‐D Salvia s.s
4686	*Salvia sclarea* L.	Europe	*Sclarea*	I‐C Salvia s.s
5207	*Salvia elegans* Vahl	America	*Calosphace*	II‐A Lasemia
5208	*Salvia patens* Cav.	America	*Calosphace*	II‐A Lasemia
5209	*Salvia pratensis* L.	Europe	*Sclarea*	I‐C Salvia s.s
6565	*Salvia candelabrum* Boiss.	Europe	*Salvia*	I‐D Salvia s.s
6566	*Salvia canariensis* L.	Europe	*Sclarea*	I‐C Salvia s.s
7956	*Salvia verbenaca* L.	Europe	*Sclarea*	I‐C Salvia s.s
8754	*Salvia hispanica* L.	America	*Calosphace*	II‐A Lasemia
8827	*Salvia tiliifolia* Vahl	America	*Calosphace*	II‐A Lasemia
8937	*Salvia columbariae* Benth.	America	*Audibertia*	II‐B/C Ramona
8982	*Salvia austriaca* Jacq.	Europe	*Sclarea*	I‐C Salvia s.s
8983	*Salvia aethiopis* L.	Europe	*Sclarea*	I‐C Salvia s.s
8984	*Salvia argentea* L.	Europe	*Sclarea*	I‐C Salvia s.s
8986	*Salvia judaica* Boiss	Europe	*Heterosphace*	Salvia s.s.
9094	*Salvia jurisicii* Kosanin	Europe	*Sclarea*	I‐C Salvia s.s
9095	*Salvia przewalskii* Maxim.	Asia	*Glutinaria*	IV‐A Glutinaria
9096	*Salvia hians* Royle ex Benth.	Asia	*Glutinaria*	IV‐A Glutinaria
9127	*Salvia farinacea* cv. *VW* Benth	America	*Calosphace*	II‐A Lasemia
9128	*Salvia nemorosa* L.	Europe	*Sclarea*	I‐C Salvia s.s
9131	*Salvia verticillata* L.	Europe	*Heterosphace*	Salvia s.s.
9132	*Salvia splendens* cv. *EB* Sellow ex. Schult	America	*Calosphace*	II‐A Lasemia
9133	*Salvia aethiopis* L.	Europe	*Sclarea*	I‐C Salvia s.s
9134	*Salvia pratensis* L.	Europe	*Sclarea*	I‐C Salvia s.s
9135	*Salvia argentea* L.	Europe	*Sclarea*	I‐C Salvia s.s
9136	*Salvia austriaca* Jacq.	Europe	*Sclarea*	I‐C Salvia s.s
9137	*Salvia farinacea* cv. BB Benth	America	*Calosphace*	II‐A Lasemia
9327	*Salvia ringens* Sm.	Europe	*Salvia*	I‐D Salvia s.s
9328	*Salvia hierosolymitana* Boiss.	Europe	*Sclarea*	I‐C Salvia s.s
9329	*Salvia virgata* Jacq.	Europe	*Sclarea*	I‐C Salvia s.s
9330	*Salvia nutans* L.	Europe	*Sclarea*	I‐C Salvia s.s
9333	*Salvia glutinosa* L.	Asia	*Glutinaria*	IV‐A Glutinaria
9335	*Perovskia atriplicifolia* *Salvia yangii*	Europe	*Perovskia*	Perovskia
9336	*Salvia pratensis* L.	Europe	*Sclarea*	I‐C Salvia s.s
9345	*Salvia greggii* A. grey	America	*Calosphace*	II‐A Lasemia
9346	*Salvia microphylla* Kunth	America	*Calosphace*	II‐A Lasemia
9347	*Salvia × jamenensis*	America	*Calosphace*	II‐A Lasemia
9349	*Salvia occidentalis* Sw.	America	*Calosphace*	II‐A Lasemia

### Floral morphometry

A subset of 24 *Salvia* accessions developed sufficient flowers to allow for morphometry of floral traits. We used the parameters defined by Benitez‐Vieyra *et al*. (2019): *lower lip length* (*lll*), *lower lip width* (*llw*), *upper lip length* (*ull*), *upper lip width* (*ulw*), *corolla tube length* (*ctl*), and *corolla tube width* (*ctw*). In addition, we introduced the parameters *stamen length complete* (*slc*), summing the lengths of connective and theca, and *style length complete* (*stlc*). For each of these accessions, we determined these parameters from a total of 30 flowers from at least two different plants. The were analysed using PCA (Rstudio, v 1.2.5033; Allaire 2012), based on the mean values over all individual measurements obtained for a given accession.

### DNA extraction

Genomic DNA from fresh leaves (60 mg starting material) from all accessions (Table 1) was isolated using the Invisorb Spin Plant Mini Kit (Stratec Biomedical), following the protocol of the manufacturer. Quality and quantity of the isolated gDNA were evaluated by spectrophotometry (NanoDrop, Peqlab), and the DNA was diluted to 50 ng μL^−1^ to be used as template for PCR.

### Primer design for B‐class genes of genus *salvia*

To specifically amplify the B‐class genes *GLOBOSA* and *DEFICIENS* from gDNA of Lamiaceae (and *Salvia* species in particular), individual primers for the respective genes were designed. To define the input for primer design, we pursued a two‐stage strategy. The closest relative to *Salvia* with a sufficiently precise annotation of flower development genes is sesame (*Sesamum indicum*). The second tool for our strategy was the annotated genome of *Salvia miltiorrhiza*, a medicinal plant that is widely used in Traditional Chinese Medicine for treatment of cerebrovascular and cardiovascular diseases (Xu *et al*. 2016). We used the annotated B‐class sequences from *Sesamum indicum* as bait for a BLAST search in the *Salvia miltiorrhiza* genome (Zhang *et al.*, 2015). The top results were double‐checked against the NCBI database. After aligning the bait sequence from *Sesamum indicum* with the homologue from *Salvia miltiorrhiza* (MEGA, v. 7.0.14, www.megasoftware.net; Kumar *et al*. 2016), we were able to design primers that flank the respective B‐class gene from its start codon to its stop codon, including all exons and introns (Table 2).

**Table 2:** Primers used in this study.

name	5′→3′	target/purpose	design
M13_fw	CGCCAGGGTTTTCCCAGTCACGAC	colony PCR and sequencing	
M13_rv	TCACACAGGAAACAGCTATGAC		
GLO1b_fw	GGGTAGAGGTAAGATTGAGATCAAG	B‐class GLOBOSA gene in Nepetoideae	current study
GLO2_rv	GAAACGCTCCTGCAGATTAGGC		current study
DEF1_fw	ATGGCTCGTGGGAAGATCCAGATC	B‐class DEFICIENS gene in Nepetoideae	current study
DEF2_rv	GCAAATGTAGTGAGGTCCGAGGC		current study
18S_fw	GCGGAGTCCTAGAAGCAACA	house keeping gene 18S	Yang *et al*. 2010
18S_rv	CTTCGGGATCGGAGTAATGA		
Ubiquitin_fw	GTTGATTTTTGCTGGGAAGC	house keeping gene Ubiquitin	Yang *et al*. 2010
Ubiquitin_rv	GATCTTGGCCTTCACGTTGT		
Actin_fw	AGGAACCACCGATCCAGACA	house keeping gene Actin	Yang *et al*. 2010
Actin_rv	GGTGCCCTGAGGTCCTGTT		
GLO_qPCR_fw	GGGTAGAGGTAAGATTGAGATCAAG	gene expression analysis of GLOBOSA	current study
GLO_qPCR_rv	TATCTTCTCCTTTCAGGTGCCT		current study

### PCR‐based cloning of *salvia* B‐class genes

We amplified the B‐class genes using genomic PCR from a 100–150 ng gDNA template. The reaction volume was 30 μL, containing 20.4 μL nuclease‐free water (Lonza, Biozym), 1‐fold Thermopol Buffer (New England Biolabs), 1 mg mL^−1^ bovine serum albumin, 200 mM dNTPs (New England Biolabs), and 0.2 mM forward and reverse primers (see primer list, Table 2), with 3 units Taq polymerase (New England Biolabs). Thermal cycler conditions for amplification of B‐class genes included initial denaturation at 95°C for 2 min; following 35 cycles at 94°C for 45 s, 58°C for 45 s, and 68°C for 2 min; ending with an extension of 68°C for 5 min. We subsequently evaluated the amplicons using agarose gel electrophoresis with NEEO ultra‐quality agarose (Carl Roth, Karlsruhe, Germany), visualizing DNA with SYBRsafe (Invitrogen, Thermo Fisher Scientific, Germany) or Midori green Xtra (Nippon Genetics Europe) upon blue light excitation on a digital gel documentation system (SafeImager; Invitrogen). The fragment size was determined using 100 bp or 1 kb size standards (New England Biolabs). We purified the amplicons using the MSB Spin PCRapace kit (Stratec) according to the manufacturer's instructions for subsequent cloning into the pGEM‐T Easy Vector System, following the protocol of the producer (Promega). After blue‐white screening according to Sambrook *et al*. (1989), white (positive) colonies were grown overnight under selective conditions (LB medium with 0.1% w/v ampicillin at 37°C and shaking at 180 rpm). After 2 h from the onset of incubation, aliquots of 1 μL were used as template for a colony PCR using the conditions described above but with the universal primer pair M13 (fw/rv) (Table 2) nested in the vector. For colonies with inserts of the correct size, cultivation continued until the following morning to isolate the inserts (Roti‐Prep Plasmid Mini kit; Carl Roth) and these were sent for sequencing (Eurofins Genomics, Ebersberg, Germany or Macrogen Europe, Amsterdam, The Netherlands) using the universal M13 primers.

### Sequence analysis

We examined quality of the obtained sequences using the software FinchTV v. 1.4.0 (Patterson *et al*. 2004) after sequencing from two directions using the M13 forward and reverse primers (Table 2). To construct alignments of the overlapping regions, we used MEGA v. 7.0.14 (Kumar *et al*. 2016) to obtain the full sequence for B‐class genes of different *Salvia* species. To detect coding regions in the full sequence, we used cDNA from European *Salvia pratensis* and American *Salvia elegans* as templates. We constructed a multiple sequence alignment of all gDNA and cDNA sequences for each gene of interest using the MUSCLE algorithm (Edgar 2004) in MEGA7. To infer coding sequences, we removed the introns *in silico* for all sequences of the *Salvia* dataset. The complete *GLO* and *DEF* sequences of *Salvia miltiorrhiza*, these were extracted from the herbal plant database (http://herbalplant.ynau.edu.cn/html/Genomes/), the sequence of *Sesamum indicum*, which served as outgroup, was obtained from the NCBI database (Johnson *et al*. 2008; Wei *et al*. 2011). The full‐length multiple sequence alignment (including exons and introns) served then as input for Bayesian inference using the BEAST package v. 1.8.4 (Drummond *et al*. 2012), by simulating Markov chains for 10 million generations, and sampling every thousandth generation. We estimated the calculation parameters using the BEAUti routine, before calculating with the BEAST routine. The integrated TreeAnnotater allowed determination of the burn‐in (10%; 1.000). For visualization of the resulting trees, we used the Java‐based software FigTree v. 1.4.2 (Rambaut 2014), choosing the posterior probabilities (PP) option to estimate the degree of significance.

### Sampling of floral organs and quantification of gene expression

Closed and open flowers of bee‐pollinated, purple‐coloured *Salvia pratensis* (indigenous in Central Europe) and hummingbird‐pollinated, red‐coloured *Salvia elegans* (originating from Central America) from plants in the collection of KIT‐JKIP were dissected into five parts (calyx/sepal, corolla/petal, ovary, stamen, and style), and immediately shock‐frozen separately in liquid nitrogen. Data represent three biological replicates for each of the two development stages (closed/open), for each of the two species. As criterion for closed flowers, we used: corolla visible but closed, flower buds of total length 1 cm in *S. pratensis*, and total length 2 cm in *S. elegans*. As criterion for opened flowers, we used: corolla open releasing the reproductive organs, flower ready to pollinate, but yellowish pollen still visible at the anthers as hallmark of recent opening. After grinding frozen tissues (50–100 mg) to a powder using a Tissue Lyzer (Qiagen), we extracted total RNA using the Spectrum™ Plant Total RNA Kit (Sigma‐Aldrich, Deisenhofen, Germany) following the manufacturer's instructions. We synthesized cDNA from 1 μg RNA template using the reverse transcriptase M‐MuLV according to Svyatyna *et al*. (2014), and determined steady‐state transcript levels of floral development genes by quantitative real‐time PCR (qPCR), performed with a CFX96 Touch™ Real‐Time PCR Detection System (Bio‐Rad Laboratories, Munich, Germany). We designed the primers for *GLO* based on the *Salvia* draft genome (Zhang *et al*. 2015), and first verified their functionality with semi‐qRT‐PCR experiments. For details on primer sequences, see Table 2. The qPCR was conducted in a reaction volume of 20 μL, with 1 μL 1:20 cDNA dilution labelled with SYBR Green. Thermal cycler conditions for qPCR included an initial denaturation at 95°C for 3 min, preceding 39 cycles with denaturation at 95°C for 15 s, annealing at 58°C for 40 s, a plate‐reading step, and synthesis at 68°C for 30 s, with a final synthesis step at 68°C for 5 min, followed by a denaturation step for 10 s at 95°C for the melting curve. The melting curve was collected through 60 steps of 5 s per step, increasing temperature by 0.5°C per step. As internal standard, we selected the housekeeping genes 18 S rRNA, Actin, and Ubiquitin (Yang *et al*. 2010). Data represent three biological replicates, each in technical triplicates. The relative expression of the floral development genes was calculated against these reference genes using the 2^−∆Ct^ method (Livak & Schmittgen 2001). We tested the significance of differences in transcript levels with a two‐tailed *t*‐test.

## RESULTS

### Floral morphology of new‐world *salvia* is more diverse than in old‐world *salvia*

**Fig. 1:**
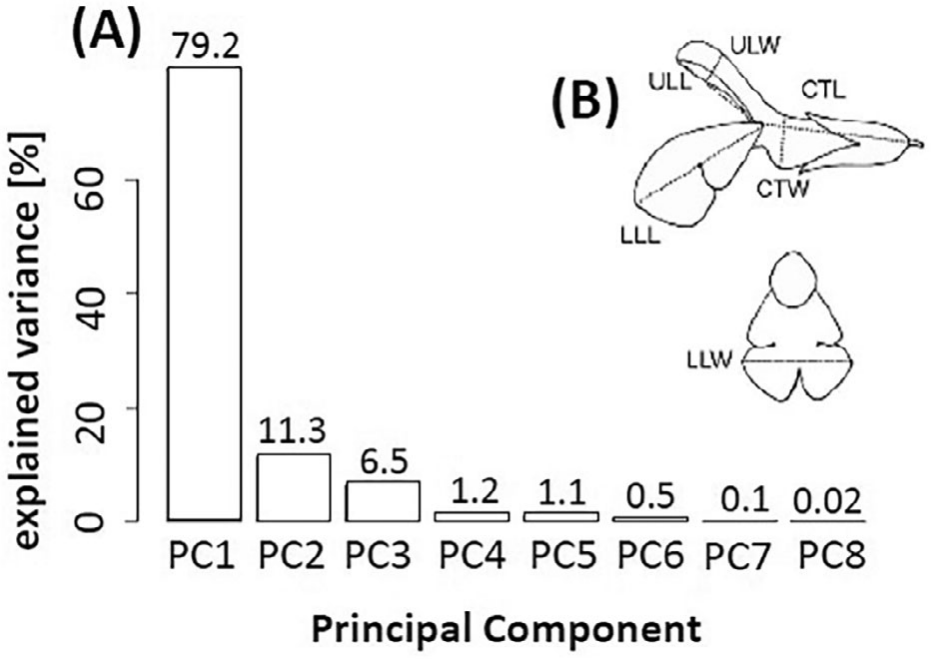
Morphometry of 42 accessions, covering 24 species of Old and New World *Salvia*. (A) Impact of principal components (PCA for measured floral traits of different *Salvia* species), with PC1 describing size and PC2 the degree of radial symmetry. (B) Definition of shape descriptors used to quantify flower morphology, based on Benitez‐Vieyra *et al*. (2019). lll: lower lip length, llw: lower lip width, ull: upper lip length, ulw: upper lip width, ctl: corolla tube length, ctw: corolla tube width. Descriptors used: stc: stamen length complete, stlc: stylus length complete.

**Fig. 2:**
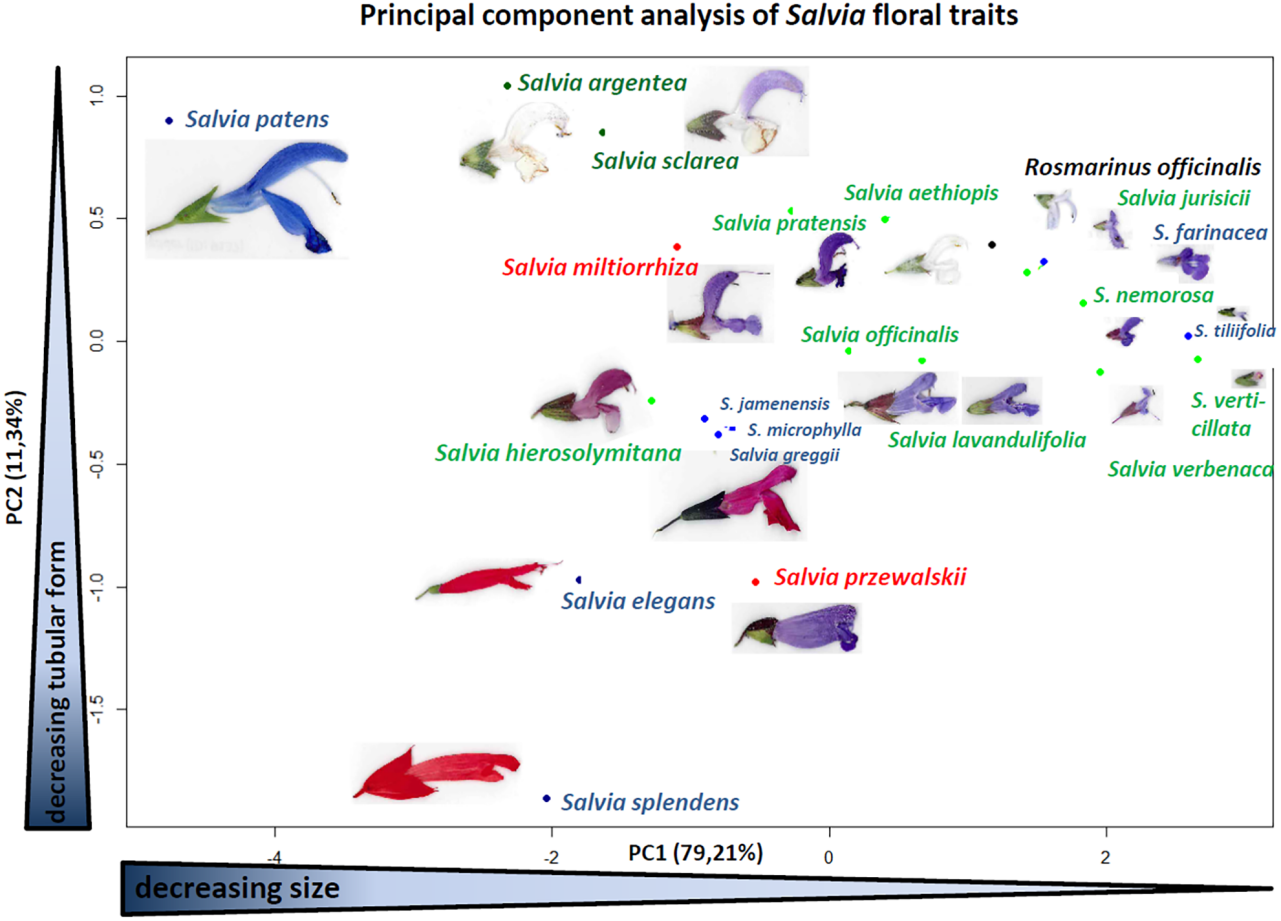
Graphical representation of the major two components defining floral morphology of *Salvia* as derived from the PCA explaining 90.55% of total variation. The dataset covers 42 accessions, representing 24 species of Old and New World *Salvia*, individual values derive from 30 individual flowers. PC1 is mainly defined by size and explains 79.21% of total variation. PC2 is mainly defined by the degree of radial symmetry and explains 11.34% of total variation. Flowers are depicted true to their relative size.

To examine the extreme differentiation of genus *Salvia*, along with remarkable variations in size and shape of flowers and floral organs – from large, 5 cm in length, vividly‐coloured blossoms (e.g., *S. patens* Cav.), to inconspicuous inflorescences, < 1 cm, which remain closed throughout development (e.g., *S. verbenaca* L.), and render this morphological diversity accessible to mechanistic analysis, we combined morphometry with PCA, measuring 660 individual flowers from 24 *Salvia* species. We used the parameter set developed by Benitez‐Vieyra *et al*. (2019): lower lip length (lll), lower lip width (llw), upper lip length (ull), upper lip width (ulw), corolla tube length (ctl), corolla tube width (ctw), complemented by the parameters stamen length complete (slc) and stylus length complete (stlc). The relative contributions of these parameters to total variability was then assessed by PCA, where PC1 explained 79%, while PC2 accounted for an additional 11% (Fig. 1). A plot of the species over these two components, representing 91% of total variability (Fig. 2), revealed a steady reduction in flower size along the *x*‐axis describing PC1, and a transformation of corolla shape from tubular with radial symmetry to dorsiventral with clear lips along the *y*‐axis describing PC2. The European species formed a diffuse cluster in the upper right part of the graph (small flowers with distinct dorsiventrality). However, *Salvia argentea* and *Salvia sclarea* represent clear outliers to this pattern. Both show clearly dorsiventral, but large, flowers, both are pollinated by carpenter bees. The closely related species *S. officinalis* and *S. lavandulifolia* (synonymous to *S. officinalis* subsp. *lavandulifolia*) cluster closely together with only a small shift in PC1, reflecting the overall slightly bigger flowers of *Salvia officinalis*. In contrast to the European species of *Salvia*, the American species do not form a cluster. A group of the three closely related species, *S. greggii*, *S. microphylla* and *S. × jamenensis*, are intermediate in size and asymmetry, positioned in the vicinity of the Eurasian *S. hierosolymitana*, while *S. tiliifolia*, with their small and dorsiventral flowers, maps near to the small‐sized European *S. verbenaca*. In contrast, *S. patens*, with its large upper lip, is located as outlier in the upper left corner of the PCA plot.

Because of its strongly elongated and round corolla tube (an adaptation to hummingbirds), *S. splendens* is positioned in the lower left quadrant of the PCA plot, where its tubular corolla is loosely grouped with *Salvia elegans* (which is also pollinated by hummingbirds), and the Asiatic *Salvia przewalskii*. Overall, the morphometric analysis reflects the pronounced morphological diversity in New World species of *Salvia* as compared to the more uniform European clade.

### *Salvia* B‐class protein *GLOBOSA* harbours the typical MIKC domains

**Fig. 3:**
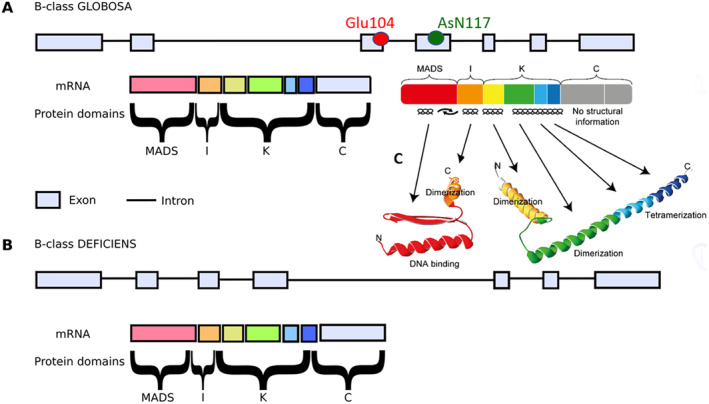
Exon‐intron structure of the B‐class genes *GLOBOSA* (A) and *DEFICIENS* (B) in *Salvia*. Exons are indicated as boxes, introns as lines, all lengths are drawn to scale. The position of the structurally relevant polymorphisms within *Salvia* are highlighted in red (position 104) and green (position 117). (C) Protein structure of the Arabidopsis E‐class homologue SEPALLATA3 from *A. thaliana* according to Käppel *et al*. (2023).

To test, whether the large morphological diversity of *Salvi*a flowers is reflected in a corresponding diversity of the B‐class factor *GLOBOSA*, we cloned and deciphered the full‐length coding sequences and the genomic sequences of all 42 accessions. These sequences were of comparable length, between 1638 bp for *S. greggii* to 1790 bp for *S. glutinosa*, including start and stop codons. The homologues from the Lamiaceae taxa used as outgroups are slightly longer, with 1851 nucleotides in *Perovskia atriplicifolia*, and 1883 nucleotides in *Dracocephalum rupestre*. Alignment with the respective mRNA revealed 7 exons and 6 introns (Fig. 3A). Around two‐thirds of the sequence are intronic. This structure reflects the characteristic protein domains of the MIKC‐MADS‐type transcription factors (Fig. 3A,C), with exon 1 encoding the MADS‐domain, exon 2 the intervening (I) domain, exons 3 to 6 the K‐domain, and exon 7 the C‐terminal domain. The intron 2, separating the coding region for the I and the K‐domain, is unusually long.

**Fig. 4:**
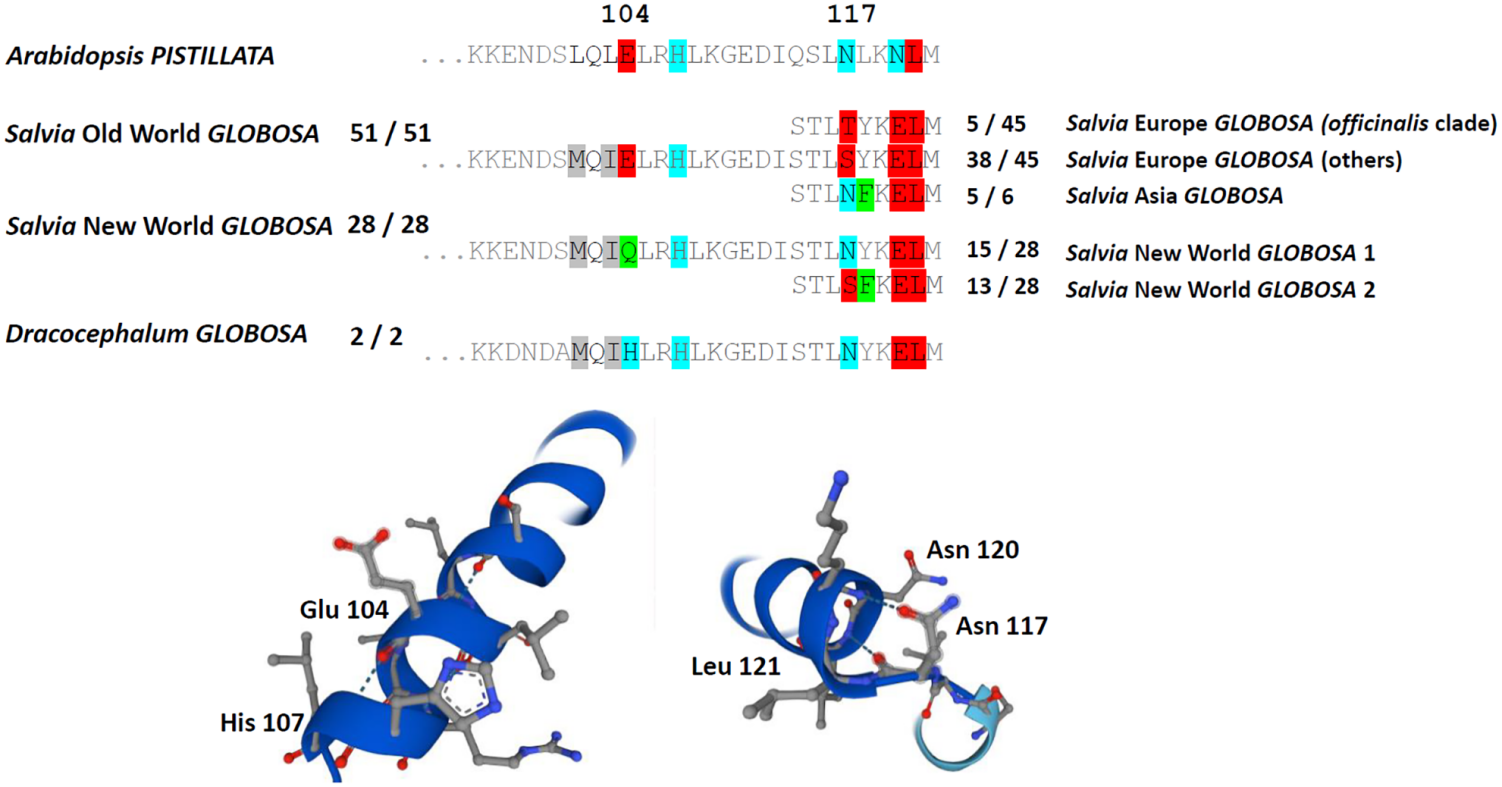
Structurally relevant amino acid exchanges in the coding sequences of GLOBOSA proteins inferred from the genomic sequences of 24 species of Old and New World *Salvia* using the Arabidopsis B‐class protein *PISTILLATA* as template.

We inferred coding sequences for *GLO* from the exons, ranging from 633 nucleotides in *Salvia elegans* to 648 nucleotides in several European species of *Salvia*, translated these *in‐silico* into amino acids, and aligned them, along with sequences from other Lamiaceae that are available in UniProt. This alignment showed a generally high level of conservation, especially in the MADS domain. In this background, two polymorphisms were important because they related to the geographic distribution of the accessions (Fig. 4). Both were located in the K‐box, responsible for interaction with binding partners. In the first subdomain, a glutamic acid (E104), strictly conserved in all 51 sequences recovered from Old‐World *Salvia*, was replaced by glutamine (Q104) in all 23 sequences recovered from New‐World *Salvia*. This glutamate residue is structurally relevant because it sustains a hydrogen bond with a histidine at position 107. Since this histidine is also conserved in New‐World *Salvia*, exchange for glutamine would disrupt this hydrogen bond.

A second polymorphism concerns position 117, the second subdomain of the K‐box. However, this geographic pattern differs: all 38 sequences from the European subgenus' *Sclarea'*/I‐C cluster show an S117 (the five sequences from the *officinalis* clade have T117, which is structurally irrelevant). In contrast, the majority (five out of six) of sequences from East Asian *Salvia* display an asparagine (N117) at this site. Interestingly, the *GLO* homologues from New‐World *Salvia GLO* diverge more‐or‐less symmetrically into the same two types: 15 out of 28 harbour the N117 characteristic for *Salvia* from East Asia, while 13 out of 28 harbour the S117 typical for the European subgenus' *Sclarea'*/I‐C cluster. Again, this polymorphism is structurally relevant, because the N117 forms two hydrogen bridges to the dyad N120 and L121. In the variant with S117, the hydrogen bridge to the highly conserved L121 can be served, but the interaction to N120 is missing and even replaced by a repulsion.

Both polymorphisms are located in that part of the K‐box participating in dimerization (Fig. 3C) and, therefore, are expected to also be of functional relevance. While the first polymorphism, at position 104, separates Old‐ from New‐World *Salvia*, the second polymorphism separates two clades of *Salvia*. One clade comprises species from East Asia and about half of the New‐World species, the other comprises species from Europe and the remaining half of the New‐World species.

### Expression of *GLOBOSA* is correlated with corolla expansion

**Fig. 5:**
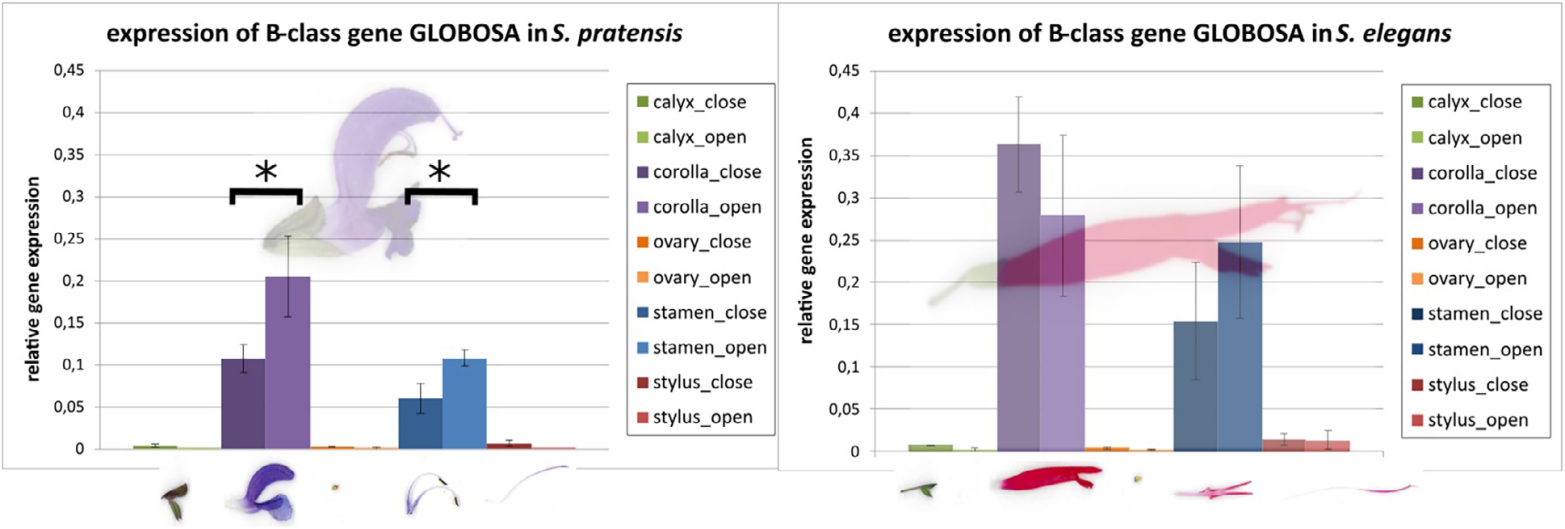
Expression pattern for the B‐class gene *GLOBOSA* in different floral organs of *Salvia pratensis* and *Salvia elegans*. Steady‐state transcript levels in the depicted organs (calyx, corolla, ovary, stamen, and style) either in closed (first bar) and opened (second bar) flowers. Error bars ±SD from three biological replicates. *Significant differences at *P* < 0.05 (*t*‐test).

The B‐class genes have been intensively studied with respect to their role in floral organ identity. The differences in the *GLO* protein from different *Salvia* species are unlikely to relate to this function in organ identity because the pattern of floral organ determination does not differ within the genus. As shown previously, it is mainly floral size and dorsiventrality where Old‐ and New‐World *Salvia* differ, i.e., traits developing *after* organ identity had already been defined. We wondered, therefore, whether *GLO* is expressed when the already differentiated organs expand into the mature flower. Comparing a pair of representatives for Old‐World and New‐World species that contrast with respect to floral morphology, we measured expression of *GLO* in the different organs, either in young and still closed, or in mature and already open flowers. *Salvia pratensis* is a purple‐coloured bee‐pollinated species from Central Europe, whereas *Salvia elegans* represents an intense red‐coloured hummingbird‐pollinated species from Central America. Flowers of both stages were dissected into calyx, corolla, ovary, stamen, and style and five organs, and steady‐state levels of *GLO* transcripts measured by qPCR. Three housekeeping genes (see Table 2) were tested in semi‐qPCRs for their suitability as reference genes in flower development, but only the 18S gene was expressed in a stable manner in all organs and all the two tested development stages. Irrespective of species, *GLO* expression was maximum in corolla and stamina (Fig. 5), while expression in calyx, ovary, and style was negligible. Thus, the pattern of expression, in the second and third whorl, is congruent with expectations for a B‐class gene. Interestingly, this expression remained high even in the opening flower, long after organ identity had been laid down. In the case of *S. pratensis*, expression was even significantly stimulated in both corolla and stamina during flower expansion. While the expression pattern was comparable between the two species, the amplitude of expression differed conspicuously, with three‐fold higher transcript levels in young flowers of *S. elegans* as compared to young flowers of *S. pratensis*. This difference diminished during flower expansion, but still remained significant, especially in the stamina (Fig. S2). Thus, there is a clear correlation between the expression of *GLO* and the expansion of corolla and stamina, both being strongly promoted in the New‐World species *S. elegans* during different stages of anthesis.

### Phylogeny of *GLOBOSA* reveals geographic supported clusters within *salvia*

We had observed structurally relevant polymorphism in the *GLO* protein that followed a distinct geographic pattern (Fig. 4). Hence, we inferred a phylogenetic tree based on the complete *GLO* region (including introns) of 38 species in total. With the exception of sequences for *Salvia miltiorrhiza*, taken from the herbal genome database, and the outgroup *Sesamum indicum* obtained from the NCBI database, the entire set of 87 sequences derived from the current study yielded an alignment of 2517 base pairs in length. At the protein level, the *GLO* protein is shorter in species from East Asia (213 aa) and the New World (214 aa) compared to the species from Eurasia (215 aa in subgenus ‘*salvia’* and 216 aa in subgenus *‘sclarea’*).

**Fig. 6:**
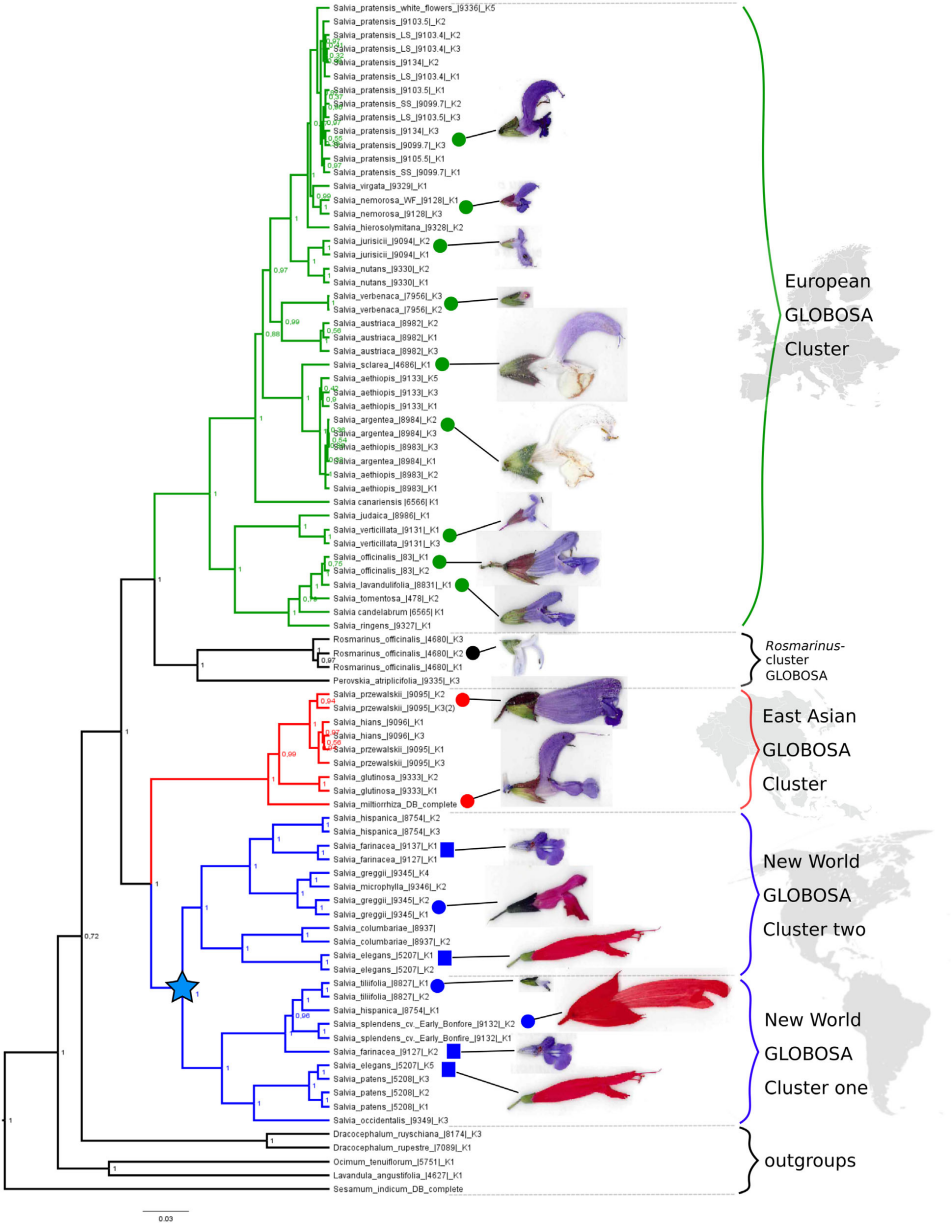
Phylogeny constructed for the genus *Salvia* based on B‐class gene *GLOBOSA* (complete sequence including exons and introns) using Bayesian inference; values at nodes indicate respective posterior probability values. Green: European *Salvia* species, blue: New World *Salvia* species, red: East Asian *Salvia* species, black: Lamiaceae taxa used as outgroups. Geographic regions are not drawn to scale. K1, K2 and K3 correspond to individually sequenced clones for the respective species. Flower morphology is shown for representative species by scale. Asterisk indicates gene duplication in the monophyletic New World *GLOBOSA* cluster.

This geographic separation is also reflected in very high (equal to 100%) posterior probability values between the monophyletic New World, East Asian, and European (including *Rosmarinus*) clusters in the phylogenetic tree (Fig. 6) inferred for the entire *GLO* sequences (including both exons and introns). The often‐reported paraphyly of *Salvia* is also seen in this tree, with *Rosmarinus* and *Perovskia* forming a defined clade within the European cluster of *Salvia*. *Sesamum indicum*, Pedaliacea, a different family within the Lamiales, as well as representatives of the genera *Ocimum*, *Lavandula* and *Dracocephalum*, sister clades to *Salvia* within the Lamiaceae, are located as outgroups and match well with the expected topology. Thus, overall, the tree reflects the known taxonomic relationships within the paraphyletic genus *Salvia*, as well as its relationship with neighbouring taxa. On the background of this phylogenetic pattern, the geographic differences become even more significant:

Most strikingly, the *GLO* phylogeny reveals two main clusters – one comprises species from the New World and East Asia, the other species from Europe and the Middle East, as well as *Rosmarinus*. This European cluster, consisting of 19 species in total (each species represented by several copies), is further subdivided into two clades that are highly supported by 100% posterior probability values. These clades match the subgenera *Salvia* and the *Sclarea* proposed by Kriebel *et al*. (2019) and Will & Claßen‐Bockhoff (2017). The smaller clade in our study, composed of *Salvia officinalis* and morphological related species, such as *S. lavandulifolia* and *S. tomentosa* (see Figs. 6 and 2, respectively), corresponds to the subgenus *Salvia* of those authors. The second subclade is more extended and comprises 11 species. It corresponds to the subgenus *Sclarea* and is subdivided into two significantly supported clusters. One cluster with *Salvia sclarea*, *S. argentea* and *S. aethiopis* is sister to a group of eight species (including *S. pratensis* and *S. nemorosa*). Thus, the phylogeny based on *GLO* reflects the integrity of European *Salvia* and, down to the details, matches the taxonomic substructures reported in the literature, supporting the notion that inferring phylogenies based on *GLO* as marker leads to meaningful results.

*Rosmarinus officinalis* and *Perovskia atriplicifolia* (also termed *Salvia rosmarinus* and *Salvia yangii*, respectively) form a distinct and statistically supported clade that is sister to the previously described European *Salvia* clade (see Fig. 6, labelled black). Thus, European *Salvia* species form a larger taxonomic unit together with *Rosmarinus*, while the representatives of other geographic regions, such as the East Asian and the New‐World *Salvia* species, constitute a separate, well‐supported cluster (see Fig. 6, labelled red and blue). Among these, the East Asian *Salvia* species form a monophyletic group that is clearly delineated from New‐World *Salvia* species.

All the above patterns match phylogenies from earlier studies (Will & Claßen‐Bockhoff 2017; Kriebel *et al*. 2019). The East Asian region is represented by four species in our study (among others the medicinal relevant *Salvia miltiorrhiza*). For the New‐World *Salvia*, we chose 10 species including economically relevant species like *Salvia hispanica*. While for the European and East Asian *Salvia* our data were more or less of a confirmatory nature. The use of *GLO* as phylogenetic marker provides novel insights into phylogenetic relationships within *Salvia* from the New World.

### Phylogeny using *GLOBOSA* reveals a duplication in new‐world *salvia*

Our study included 10 species, each represented by several accessions from the New‐World *Salvia*. These species were separated from East Asian and even more distant from the European species (blue in Fig. 6). This New World cluster is divided into two clear clusters with a separation that is supported by 100% posterior probability values. For the sake of clarity, in the displayed phylogenetic tree, we introduce designations for the two distinct New World clades. The lower American cluster is termed New World *GLO* cluster 1, whereas the upper American cluster is New World *GLO* cluster 2 (see labels in Fig. 6). Interestingly, for three species (*S. elegans*, *S. hispanica*, and *S. farinacea*), we found *GLO* alleles located in both clusters (highlighted by asterisks in Fig. 6). Both clusters were structured into two smaller clades. Cluster 1 includes species that had been delineated as core subgenus *Calosphace* by Kriebel *et al*. (2019), such as *S. splendens* and *S. hispanica*, and delineated from the non‐core *Calosphace* (such as *S. elegans* and *S. columbariae*). Thus, the phylogenetic topology of the New World *GLO* clusters further differentiates the topologies found in earlier studies, supporting a model of two independent B‐class *GLO* genes that are confined to the New World. Despite a larger sample volume, no comparable subdivision was observed in Eurasian *Salvia* species, favouring the conclusion that this subdivision must have occurred *after* the arrival of *Salvia* in the New World. This is supported by the fact that most New‐World *Salvia* species contain both GLO loci.

### Phylogeny using *DEFICIENS* reveals a duplication in both old and New World *salvia*

We wondered whether the duplication of *GLO* in New‐World *Salvia* would be reflected in a duplication of its binding partner *DEF*. Therefore, we also cloned and sequenced the B‐class gene *DEF*. Typical MIKC‐type domains could be detected, such that these *DEF* homologues appear very similar to their *GLO* counterpart, including a gene structure with seven exons and six introns (Fig. 3B). Here, intron 4, separating the coding regions of the dimerization and tetramerization subunits of the K‐domain, turns out to be of unusual length. When the inferred exons are translated *in silico*, gene products between 225 amino acids (in *Rosmarinus officinalis*) and 241 amino acids (in an allele of *Salvia patens*) are predicted.

The amplification of this gene was more difficult compared to that of *GLO*, such that this dataset comprises a reduced number of accessions and species as compared to the *GLO* study. Nevertheless, even this smaller dataset revealed a clear pattern for *DEF* in the phylogenetic analysis (Fig. S1). There is a clear and significant split into two distinct clusters, supported by 100% posterior probability values. The individual clones derived from a single amplicon often led to sequences that diverge into the different clusters, e.g., *Salvia pratensis* ID9134 K3 in the upper and K4 in the lower cluster, or *Salvia elegans* ID5207 K3 in the upper and K2 in the lower cluster (Fig. S1). Thus, there are two *DEF* loci in *both* Eurasian and New World species of *Salvia*, in contrast to the situation in *GLO*, where such a duplication was seen only for *Salvia* from the New World.

## DISCUSSION

The morphological species concept traditionally used for classical taxonomy was later replaced by a genetic concept, where a species is defined as a group of individuals that are able to entertain gene flow (Mayr 1942). While the much stricter propagation barriers of metazoan animals are well suited for such a genetic species concept, it seems less powerful when applied to plant speciation. Genetic phenomena such as allopolyploidy support breaches of propagation barriers. Hybrid sterility can be bridged by vegetative propagation until fertility is restored by genetic adjustments, such as genome duplication. Moreover, gene flow is shaped by interaction with non‐plant organisms, such as pollinators. These phenomena, to a certain extent, challenge the genetic species concept for plants. Rather than considering species as defined and separate entities, it might be more fruitful to focus on the processes leading to these entities. In other words: we should describe plant speciation rather than plant species. Genetic factors that redirect evolutionary change will lead to a self‐amplifying divergence. Since gene flow of Angiosperms is strongly dependent on pollinators, genetic changes leading to altered floral morphology might qualify as such genes of speciation. We pursued this idea for *Salvia*, because, in this genus, speciation has obviously been extremely active. Moreover, a morphological innovation, the lever structure, has been shown to be a driver for speciation (Claßen‐Bockhoff *et al*. 2004). We used the shift from mostly *Hymenoptera*‐pollinated Eurasian *Salvia* to bird‐pollinated New‐World *Salvia* as framework to link speciation with the B‐gene *GLOBOSA*, known to control the definition of petals that convey the function of pollinator attraction. The current study mainly asked whether *GLO* can be associated with the diversification of New‐World *Salvia*. The results can be discussed via three main questions: Can morphological diversity be assigned to specific morphogenetic traits? Does the expression pattern of B‐genes help to explain morphological differences? Does the B‐gene *GLO* qualify as a gene of speciation?

### Morphology matters – How genetic events are reflected as morphogenetic processes

We first asked whether the floral diversity of *Salvia* can be assigned to specific morphogenetic processes, or whether flowers in this genus exhibit a general variability. Since a deeper analysis of the more than 1000 known species of *Salvia* would be an insolvable task, we selected a set of species representing utmost diversity with respect to geographic origin, flower characteristics, or pollination behaviour. This sampling strategy seemed feasible, even for the smaller group of New‐World *Salvia* species, since the PCA of the morphometric parameters displayed a spread over the entire area of the plot, reflecting the overall higher variation in size and shape for New‐World *Salvia* species as compared to the more abundantly represented Eurasian *Salvia* species (see Fig. 2).

While morphometric analyses of *Salvia* corollas have been conducted in the past, for South African ornithophilous species (Wester & Claßen‐Bockhoff 2006), and for melittophilous species from the Middle East (Kharazian 2012), we included stamen and style length as additional parameters relevant for pollination specificity, and employed the morphometric parameter set developed by Benitez‐Vieyra *et al*. (2019). Although those authors also analysed New‐World *Salvia*, the species set was completely different from that of the current study (only *Salvia elegans* was addressed in both studies), which also included a large number of Old‐World *Salvia*. The large geographic range is accompanied by an extended morphogenetic space. On this background, the fact that almost 85% of the variability can be assigned to only two morphogenetic parameters (corolla length and radial symmetry) shows that floral diversity is not due to an overall variable morphogenesis (that genetically would be caused by variation in numerous loci). Instead, this floral diversity is linked to a specific morphogenetic process, increasing the chance that they can be linked to polymorphisms in specific genes.

While floral traits of European *Salvia* cover a relatively small region of the morphospace, geometries of New‐World *Salvia* are far more variable, both in size and shape. For instance, pronounced dorsiventrality is seen in *S. patens*, while *S. elegans* exhibits almost perfect radial symmetry. This expansion of the morphospace might be linked to a shift in the co‐evolutionary path, from *Hymenoptera* towards hummingbirds. The close connection between floral morphology and pollination strategy is illustrated by cases where flowers are morphologically similar but geographically and, therefore, phylogenetically, distant. For instance, the Old‐World species *S. hierosolymitana* and the New‐World species *S. greggii* are nested together in the PCA and, if grown in Europe, are both pollinated mostly by large bees (in the New World for *S. greggii*, the large bees are replaced by hummingbirds). Conversely, the Old‐World *S. jurisicii* and the New‐World *S. farinaceae* are neighbours at a different location on the PCA plot, and both are visited by smaller bee species. Thus, it seems to be geometry that shapes gene flow. Genes that alter floral morphology might, therefore, qualify as genes of speciation.

### Beyond organ identity – Expression of *GLOBOSA* correlates with organ size

The B‐genes such as *GLO* are classically assigned to organ identity in the central two whorls, i.e., petals and stamina (Bowman *et al*. 1991; Coen & Meyerowitz 1991). This function would require expression very early in flower morphogenesis, namely, when the primordia are laid down in the newly committed floral meristem. We found that *GLO* transcripts accumulate much later during anthesis (Fig. 5), at a time, when floral organs already expand. Thus, this gene must convey a second function, in addition to its well‐established role for primordial differentiation. As expected from the B‐gene, expression was confined to corolla and stamina for both species: the European, bee‐pollinated *S. pratensis* and the American, hummingbird‐pollinated *Salvia elegans*. However, expression levels were significantly higher in *S. elegans*, correlating with larger organ sizes in this species. The temporal pattern of expression seems to be correlated with the progression of organ expansion. While in *S. pratensis*, expression of *GLO* in both corolla and stamina increased when the flower opened; for *S. elegans* the peak of expression in the corolla was already reached when the flowers were still closed and decreased later. In contrast, expression continued to increase in the stamina of *S. elegans* during flower opening. Considering that the whorl producing the stamina is laid down later than the whorl bearing the corolla, it is straightforward to interpret the pattern seen in *S. elegans* as a transient induction, which occurs earlier in the corolla, later in the stamina. This precocious induction in the corolla seems absent in *S. pratensis*, which might explain the smaller corolla size. The second function of *GLO* beyond organ identity might be a stimulation of cell expansion. A testable implication for this hypothesis would be that species with a long corolla are expected to express *GLO* at higher amplitude and earlier in floral development, compared to species with a shorter corolla. In this context, the extensive introns of *GLO* are interesting, especially the unusually long intron 2. First, regulatory functions have been demonstrated for the second intron of the C‐class gene *AGAMOUS* in *Arabidopsis thaliana* (Deyholos & Sieburth 2000). Second, the length of these introns is variable to an extent that they can be used as markers for population genetics, which is the topic of a forthcoming study.

A second function of *GLO*, stimulation of corolla expansion, is suggested by the expression pattern. However, such a second function would require interaction with transcriptional regulators that differ from those defining organ identity. This interaction might differ between different clades of *Salvia*. In fact, we find two amino acid polymorphisms that correlate with geographic and, thus, with morphological patterns. Both are located in the K‐box, which is crucial for dimerization. In contrast, the N‐terminal MADS domain, responsible for DNA binding (for a review see Käppel *et al*. 2023), and the intervening domain, also participating in DNA binding (Lai *et al*. 2021) are highly conserved. Both modifications in the K‐box are structurally relevant because hydrogen bonds essential for secondary structure are eliminated or even exchanged for repulsive forces. For the polymorphism at position 117, a straightforward scenario would assume that this mutation arose in East Asian *Salvia* and from there immigrated into a part of the New‐World *Salvia*. In contrast, the polymorphism at position 104 seemed to have arisen *de novo* after the arrival in the New World. Thus, a modulated interaction with binding partners might contribute to the changed floral morphology.

So far, studies of floral gene expression in Lamiaceae have been limited on metabolic pathways, such as the monoterpene synthesis genes responsible for accumulation of linalool and lavandulyl acetate in lavender (Li *et al*. 2021), or the circadian fluctuation of monoterpene synthase connected with a higher abundance of essential oils in the afternoon (Seira *et al*. 2023). Our finding that amplitude and temporal pattern of *GLO* relate to flower morphology indicates that a systematic expression study on floral organ identity markers might be rewarding for a deeper understanding of *Salvia* evolution. Our findings extend observations in *Callicarpa americana* (Alhindi & Al‐Abdallat 2021) and lavender (Wells *et al*. 2020), where expression of the *GLO* and *DEF* homologues was proceeding during late anthesis. However, since neither study separated floral organs, it is not clear to what extent these patterns are linked with corolla expansion.

### Origin and dispersal of *salvia* seen through the B‐class lens

The comparative analysis of *GLO* across *Salvia* from different continents revealed two features that contribute to the ongoing debate on the geographic origin of the genus. First, the GLOBOSA proteins from Eurasian *Salvia* are longer than those from East Asia and the New World. Second, the *GLO* gene from New‐World *Salvia* has undergone a duplication.

The origin of the genus *Salvia* has been proposed to lie either in the Mediterranean region (Will & Claßen‐Bockhoff 2017) or in Southwest Asia (Kriebel *et al*. 2019), both dated to the early Oligocene around 32 million years ago (Will & Claßen‐Bockhoff 2017; Kriebel *et al*. 2019). Considering the polymorphisms in protein length, principally, two scenarios are conceivable. *Salvia* might have originated in East Asia and acquired additional codons while spreading to Europe (whereby subgenus *salvia* with 215 amino acids would be a precursor of subgenus *sclarea* with 216 amino acids). In parallel, the genus managed to reach the New World, for instance through Beringia, which during the Oligocene served as migration route for many plant taxa living in boreo‐tropical forests (Wen *et al*. 2016). Alternatively, the long version of European GLO represents the ancestral state which became shorter during the migration to East Asia and the New World.

Our finding that the length of the GLO protein is conserved throughout both subgenera of New‐World *Salvia*, *Calosphace* (represented in our study by *Salvia elegans* and *Salvia hispanica*), and *Audibertia* (represented by *Salvia columbariae*) supports a scenario where all New‐World *Salvia* derive from a single ancestor that managed to reach the New World (Kriebel *et al*. 2019). This scenario is strongly supported by the finding of fossil pollen of an *Audibertia*‐type *Salvia* in Alaska that is dated to around 20 mya, i.e., the upper Miocene (Emboden 1964). These events predate the fossil records for the Eurasian subgenera *Salvia*, *Sclarea* and *Heterosphace*, attributed to the mid‐Miocene, only 10 mya, and thus, around 10 million years later (Will & Claßen‐Bockhoff 2017). The most straightforward model would interpret the shortest (213 amino acids) East Asian version of GLO as close to the ancestral state, from which the only one amino acid longer New World version arose early during the migration through Beringia in the late Oligocene (around 24 mya), while a second, much later spread (10 mya) from East Asia to Europe was accompanied by the longer alleles found in the Eurasian subgenera *Salvia*, *Sclarea* and *Heterosphace* supporting a model suggested by Will & Claßen‐Bockhoff (2017).

Duplication of GLO in New‐World *Salvia* is manifest, for instance, in the sequence data obtained from *S. elegans*, *S. hispanica* and *Salvia farinacea*. Here, the two *GLO* homologues cluster to different clades within the New‐World *Salvia* (see Fig. 6). In contrast to these species from the New World, for none of the 13 sequences collected from various individuals of the European species *Salvia pratensis*, nor for any of the 34 sequences obtained from other European *Salvia* species, were there any indications for a duplication. As a result, the *GLO* tree for the European species is monophyletic. This finding strongly supports a duplication event that was geographically restricted to the New World. Our result is congruent with a recent transcriptome analysis of *Salvia*, which was, however, restricted to genes expressed in roots and leaves (Hu *et al*. 2023), while flowering genes such as *GLO* were excluded.

### Was the duplication of *GLOBOSA* a driver for the diversity of New World *salvia*?

During the evolutionary history of the MIKC‐type gene family, several duplication events occurred, followed by sub‐functionalization, which resulted in functional diversification of MIKC‐type genes (Theissen & Saedler 2001). As an example, several duplications of MIKC‐type genes in wheat (*Triticum aestivum* L.) led, among other effects, to sub‐functionalization in the context of stress response to abiotic and biotic factors (Schilling *et al*. 2020). In a recent study, the functional relevance of B‐class gene duplication was uncovered for the phenomenon of heterostyly. Analysis of two paralogs of GLOBOSA in *Primula* revealed sub‐functionalization after gene duplication (Huu *et al*. 2020). While the duplication of *GLO* in *Salvia* might have unexpected implications on phylogenetic reflections of the genus, more importantly our results provide one possible genetic explanation for the vast species richness of New‐World *Salvia*.

**Fig. 7:**
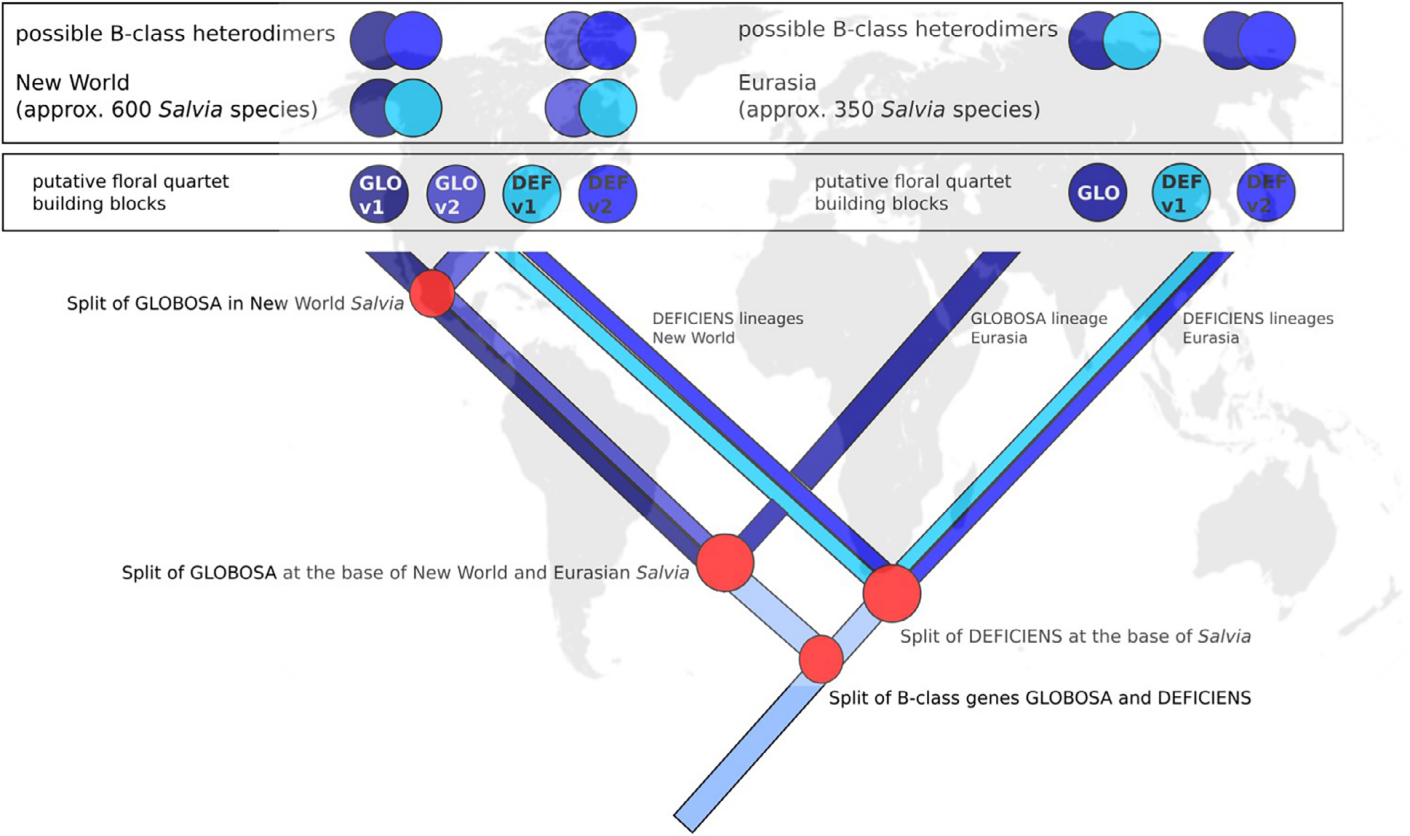
Working model for the history of B‐class genes in *Salvia* explaining the extensive floral diversity in New World *Salvia* by a duplication of *GLOBOSA*. Red circles are putative duplication events. Blue lines are different B‐class gene lineages. Blue circles is B‐class MIKC‐type MADS domain proteins. Assembling of those proteins into heterodimers and the ensuing number of possible interactions are also illustrated.

Our model (Fig. 7) is based on the finding that GLO and DEF proteins form, with only very few exceptions, heterodimers (Melzer *et al*. 2014). A phylogeny based on *DEF* (Fig. S1) shows two well‐separated clades that both include *Salvia* species from different geographic regions, pointing towards an early gene duplication of *DEF* for the entire genus *Salvia*. While such a duplication has also been found for other Lamiales, such as *Verbena* and *Mimulus*, *Salvia* seems to be the only case known so far where this has happened within the Lamiaceae (Aagaard *et al*. 2005), and our study can confirm this early duplication. For the heterodimer partner GLO, the current study shows evidence for a duplication that occurred later, because it is found exclusively in *Salvia* from the New World (Fig. 6). We propose (Fig. 7) that the duplication of *GLO* enabled neo‐ or sub‐functionalization driving the floral diversification during the spread into the New World. The number of possibilities for hetero‐dimerization with *DEF* increases by a factor of two, and the additional interaction possibilities of floral quartet complexes, in particular, the altered tetrameric interactions for A‐ and E‐class genes in the corolla (AEBB), and C‐ and E‐class genes (CEBB) in the stamina, might allow for differential morphogenesis of those organs, since we can show the expression of *GLO* concomitant with and correlated to organ expansion (Fig. 5). The extended toolbox for interaction with different downstream targets might have enabled the observed increase of floral traits, especially in the corolla (Fig. 2). In *Primula*, the GLO1 paralog facilitates the more “ancestral” gene function as initiator of corolla and stamen identity, whereas GLO2 regulates tissue growth by promoting cell expansion in the petals and stamens (Huu *et al*. 2020). The duplication of *GLO* in New‐World *Salvia* might have been the driving force behind the expansion of floral diversity and, thus, the species richness of the genus seen in the New World. To support our model, future research should focus on protein–protein interaction to validate the putative sub‐functionalized GLO and DEF monomers in New‐World *Salvia in vivo* (e.g., by Y2H). Further investigations could also focus on the potential downstream targets of the different GLO‐containing floral quartet. Such an approach, along with transcriptomic analysis of corolla and stamen development in Old‐ versus New‐World *Salvia*, could further illuminate the mechanisms of floral development, evolution, and species diversity in this genus.

## AUTHOR CONTRIBUTIONS

PN and SW contributed to the idea, topic, background information. Experimental planning and experiments were carried out by SW. SW and PN wrote the manuscript.

## CONFLICT OF INTEREST STATEMENT

The authors declare no competing financial or non‐financial interests.

## Supporting information


**Fig. S1.** Phylogeny constructed for the genus *Salvia* based on B‐class gene *DEFICIENS* (entire sequences including exons and introns) using Bayesian inference; values at nodes indicate the respective posterior probability values. Green: European *Salvia* species, blue: New World *Salvia* species, red: East Asian *Salvia* species, black: Lamiaceae taxa used as outgroups.

**Fig. S2.** Comparison of steady‐state transcript levels in developing corolla and stamen in *Salvia pratensis* and *Salvia elegans* at different stages of anthesis (adapted from Fig. 5). Significant differences are labelled, *P < 0.05, **P < 0.01 (*t*‐test).

## Data Availability

Sequence data have been uploaded to GenBank.
