# Supplementary figures and images for: B‐class gene GLOBOSA – a facilitator for enriched species diversity of Salvia in the New World?
Source: Plant Biol (Stuttg). 2025 Feb 18;27(3):333–46. doi: 10.1111/plb.70002 (PMC11950904; doi:10.1111/plb.70002)

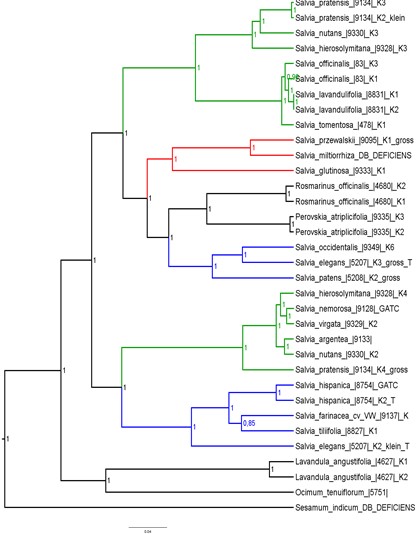

Supplement: Supplementary file 1 — Fig. S1. Phylogeny constructed for the genus Salvia based on B‐class gene DEFICIENS (entire sequences including exons and introns) using Bayesian inference; values at nodes indicate the respective posterior probability values. Green: European Salvia species, blue: New World Salvia species, red: East Asian Salvia species, black: Lamiaceae taxa used as outgroups. [file PLB-27-333-s001.jpg]

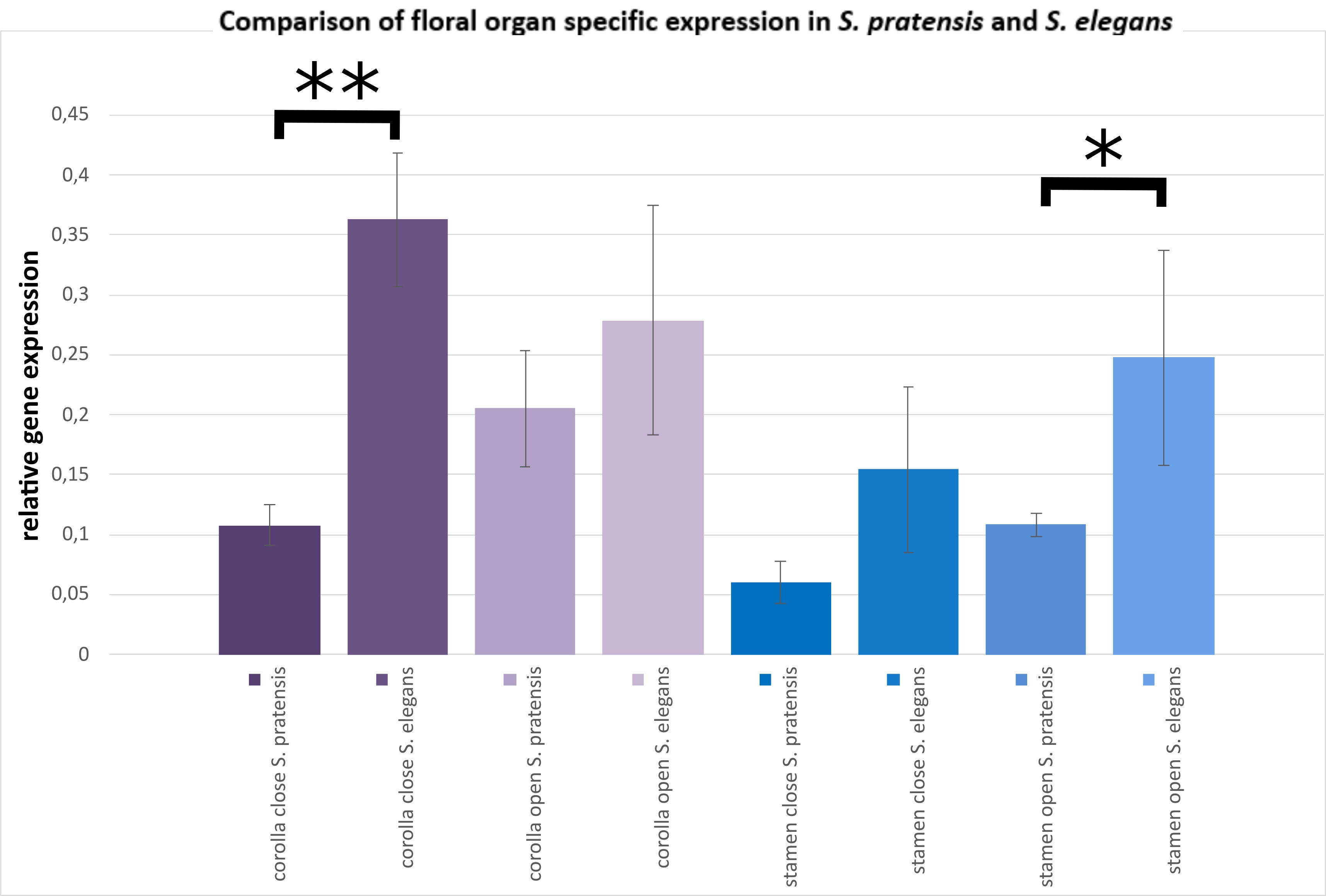

Supplement: Supplementary file 2 — Fig. S2. Comparison of steady‐state transcript levels in developing corolla and stamen in Salvia pratensis and Salvia elegans at different stages of anthesis (adapted from Fig. 5). Significant differences are labelled, *P < 0.05, **P < 0.01 (t‐test). [file PLB-27-333-s002.jpg]
